# Analysis of the Influence of Jaw Periosteal Cells on Macrophages Phenotype Using an Innovative Horizontal Coculture System

**DOI:** 10.3390/biomedicines9121753

**Published:** 2021-11-24

**Authors:** Fang He, Felix Umrath, Christiane von Ohle, Siegmar Reinert, Dorothea Alexander

**Affiliations:** 1 Department of Oral and Maxillofacial Surgery, University Hospital Tübingen, 72076 Tübingen, Germany; fang.he@med.uni-tuebingen.de (F.H.); felix.umrath@med.uni-tuebingen.de (F.U.); siegmar.reinert@med.uni-tuebingen.de (S.R.); 2 Department of Conservative Dentistry and Periodontology, University Hospital Tübingen, 72076 Tübingen, Germany; Christiane.von_Ohle@med.uni-tuebingen.de

**Keywords:** jaw periosteal cells, mesenchymal stem cells, macrophage polarization, horizontal coculture, immunomodulation

## Abstract

Jaw periosteum-derived mesenchymal stem cells (JPCs) represent a promising cell source for bone tissue engineering in oral and maxillofacial surgery due to their high osteogenic potential and good accessibility. Our previous work demonstrated that JPCs are able to regulate THP-1-derived macrophage polarization in a direct coculture model. In the present study, we used an innovative horizontal coculture system in order to understand the underlying paracrine effects of JPCs on macrophage phenotype polarization. Therefore, JPCs and THP-1-derived M1/M2 macrophages were cocultured in parallel chambers under the same conditions. After five days of horizontal coculture, flow cytometric, gene and protein expression analyses revealed inhibitory effects on costimulatory and proinflammatory molecules/factors as well as activating effects on anti-inflammatory factors in M1 macrophages, originating from multiple cytokines/chemokines released by untreated and osteogenically induced JPCs. A flow cytometric assessment of DNA synthesis reflected significantly decreased numbers of proliferating M1/M2 cells when cocultured with JPCs. In this study, we demonstrated that untreated and osteogenically induced JPCs are able to switch macrophage polarization from a classical M1 to an alternative M2-specific phenotype by paracrine secretion, and by inhibition of THP-1-derived M1/M2 macrophage proliferation.

## 1. Introduction

The success of bone tissue engineering constructs depends on the immune reaction of the recipient’s body. Since mesenchymal stem cells (MSCs) have powerful immunomodulatory potential, they are able to regulate the innate immune system [1,2]. MSCs do not only maintain tissue homeostasis by supporting the functions of connective tissue cells, and by interacting with hematopoietic progenitors, but they also regulate many types of innate immune cells, such as macrophages and dendritic cells at the microenvironmental site of inflammation [1,3,4,5]. Jaw periosteal cells (JPCs) show MSCs characteristics and have a high osteogenic potential and good accessibility. Thus, they are considered as a promising cell source for regenerative therapies in oral and maxillofacial surgery. Since JPCs are, like all other MSC types, multipotent, the desired differentiation lineage should be predefined before transplantation into the bone defect site. Therefore, the examination of interactions between osteogenically induced JPCs and immune cells is essential. Our previous studies demonstrated that untreated and predifferentiated JPCs can partially inhibit dendritic cell maturation in a transwell coculture system and regulate macrophages differentiation in a direct contact coculture system [6,7].

Macrophages have the ability to ingest and process foreign materials, dead cells debris and recruit other types of immune cells contributing to the host reaction against infection and injury [8]. An important feature of macrophages is their ability to rapidly change their phenotype in response to different microenvironmental signals, which is called macrophage polarization [9]. Classically activated macrophages (or M1 phenotype) and alternatively activated macrophages (or M2 phenotype) are two typical polarization states of macrophages. In general, M1 macrophages are characterized by the production of proinflammatory cytokines promoting T helper type 1 (Th1) responses, while M2 macrophages are involved in tissue remodeling, immune regulation supporting Th2-associated effector functions [8]. The M2 terminology also encompasses subtypes, specified as M2a, M2b, M2c and M2d macrophages, based on their distinct gene and protein expression profiles, which are induced by different cytokines.

Osteal macrophages adjacent to osteoblasts are involved in bone formation and homeostasis [10], so the successful development of biological bone scaffolds cannot be achieved without the involvement of macrophages. Due to their influence on bone formation and immune defense, the interactions between macrophages and JPCs for bone tissue engineering purposes need to be analyzed in detail. Previously, we studied the influence of JPCs on THP-1-derived macrophages in direct contact [6]. In order to analyze whether the underlying mechanism of regulation is based not only on cell–cell interactions but also on soluble factors secreted by JPCs, we used an innovative horizontal coculture system in this study. The advantage of this system, compared to the conventional transwell coculture system, is that cocultured cells can be visualized at the same time and in the same quality, because separate chambers are constructed in a mirror opposite manner. Based on these conditions, we were able to analyze paracrine effects of untreated and osteogenically induced JPCs on macrophage polarization in the horizontal coculture system.

## 2. Materials and Methods

### 2.1. THP-1 Cells and JPCs Expansion

THP-1 cells were delivered from the American Type Culture Collection (ATCC) and expanded in an RPMI 1640 medium (Thermo Fisher Scientific, Waltham, MA, USA) containing 10% heat-inactivated FBS (Sigma-Aldrich, Darmstadt, Germany), 1% penicillin/streptomycin (Lonza, Basel, Switzerland), 1% amphotericin B (Biochrom AG, Berlin, Germany) and 0.05 nM 2-mercaptoethanol (Sigma-Aldrich, Darmstadt, Germany).

JPCs from 3 donors (age: 20–31 years old) were included in this study after the approval of the local ethics committee (No. 618/2017BO2). JPCs were passaged by TrypLE-Express (Thermo Fisher Scientific, Waltham, MA, USA), and were cultured and expanded in a DMEM/F12 medium (Thermo Fisher Scientific, Waltham, MA, USA) containing 5% hPL (provided by the Institute for Clinical and Experimental Transfusion Medicine of the University Hospital Tübingen), 1% penicillin/streptomycin (Lonza, Basel, Switzerland) and 1% amphotericin B (Biochrom AG, Berlin, Germany). In addition, the mineralized performance of JPCs from 3 donors was verified through osteogenic induction experiments in a 6-well plate. Briefly, JPCs were treated with a 10% hPL DMEM-F12 osteogenic medium (containing 100 µM L-ascorbic acid) (Sigma-Aldrich, Darmstadt, Germany) and 10 mM β-glycerophosphate (PanReac AppliChem, Darmstadt, Germany) for 14 days and mineralization detection was performed by alizarin red staining. JPCs treated with a 10% hPL DMEM-F12 medium served as a control for undifferentiated cells (Figure S1).

Both JPCs and THP-1 cells were expanded in 25 or 75 cm^2^ flasks at 37 °C in a 5% CO_2_-humidified incubator and the medium was changed every two days.

### 2.2. Co-Culturing of Macrophages and JPCs in the Horizontal Coculture System

The horizontal coculture plate (Interactive Co-Culture system or UniWells^TM^), containing filters (0.6 µm) and the adapter (96-well plate size) were purchased from Ginreilab Inc. (Uchinada, Japan). Each coculture plate contains the following components: main chamber A, main chamber B, cover, common cover, O-ring and adapter. 

**Figure 1:**
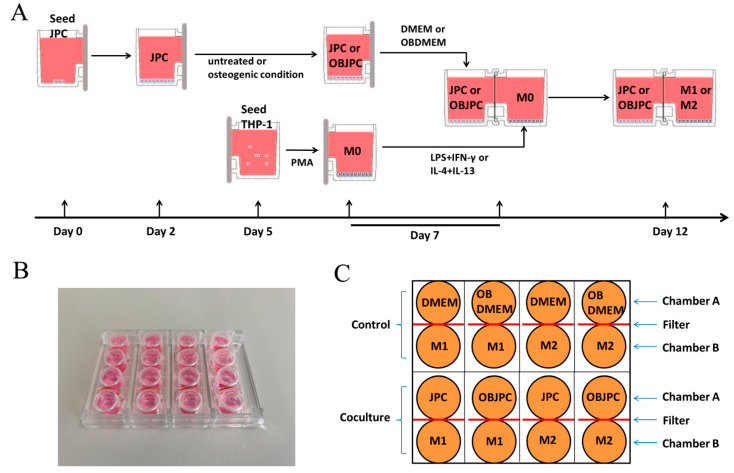
The coculture procedure and the examined groups. (**A**): Flow chart of pre- and co-culturing of JPCs/OBJPCs and M1/M2 macrophages groups. (**B**): The assembled co-culture plate was placed into an adapter. (**C**): Layout of the performed experimental groups.

The flowchart of the coculture is shown in Figure 1A. Before starting the coculture, the main chamber A or B, cover and common cover were assembled to conduct firstly the single culture of JPCs or THP-1 macrophages. 

For the pre-culturing of JPCs, cells of passage 3 (2 × 10^4^ cells/chamber) resuspended in 1.5 mL of 10% hPL DMEM/F12 medium were seeded into a chamber (chamber A, precoated with vitronectin, Gibco, Waltham, MA, USA) at day 0. After 48 h of expansion, JPCs were further cultured under untreated condition (DMEM: DMEM/F12/10% hPL) or osteogenic condition (OBDMEM: DMEM/F12/10% hPL, 100 μM L-ascorbic acid and 10 mM β-glycerophosphate) in separate chambers for 5 days. Medium change was performed every other day. For the control group, 1.5 mL JPCs-free DMEM/F12 complete medium (with or without osteogenic stimuli) was added into a parallel coated chamber following the same medium change steps. Here, we define the used abbreviations in Table 1.

For the pre-culturing of THP-1 macrophages, THP-1 cells of passage 10 (4 × 10^5^ cells/chamber) resuspended in 1.5 mL RPMI 1640/5% hPL medium were seeded into a chamber B at day 5. Phorbol-12-myristate-13-acetate (5 nM PMA, Sigma-Aldrich, Darmstadt, Germany) was used to induce M0 differentiation of THP-1 cells cultured within chamber B for 48 h. 

At day 7, the coculture of JPCs/OBJPCs and M1/M2 macrophages was started by assembling chambers A/B. The assembled coculture chambers were set into an adapter and incubated for further 5 days (Figure 1B). For M1 macrophages differentiation, THP-1 macrophages were cultured in RPMI1640/5% hPL medium containing 15 ng/mL lipopolysaccharide (LPS, Sigma-Aldrich, Darmstadt, Germany) and 20 ng/mL interferon-γ (IFN-γ, Sigma-Aldrich, Darmstadt, Germany). Differentiation to M2 macrophages was induced in RPMI1640/5% hPL medium containing 20 ng/mL interleukin 4 (IL-4, Sigma-Aldrich, Darmstadt, Germany) and 20 ng/mL interleukin 13 (IL-13, Sigma-Aldrich, Darmstadt, Germany).

JPCs/OBJPCs and M1/M2 macrophages cocultures as well as control groups were cultured for further 5 days. Day 12 was the examination time point for flow cytometric or gene and protein expression analyses. Figure 1C shows the analyzed control and coculture groups.

In order to analyze the secretion of osteogenically induced JPCs in the coculture system, we also performed monoculture experiments with OBJPCs. Specifically, the OBDMEM and the RPMI1640/5% hPL media were set as the medium control groups; OBJPCs cocultured with the RPMI1640/5% hPL medium was set as the unstimulated (without macrophagic stimulation) OBJPCs control group; OBJPCs coculture with macrophages-free RPMI1640/5% hPL medium containing LPS and IFN-γ was set as the OBJPCs-M1RPMI experimental group; OBJPCs cocultured with macrophages-free RPMI1640/5% hPL medium containing IL-4 and IL-13 was set as the OBJPCs-M2RPMI experimental group. The supernatants from the chamber B were collected after 5 days of coculture and protein secretion was subsequently measured using a human cytokine proteome profiler array (Figure S3A).

In addition, we compared the diffusion capacity of the used horizontal coculture system by comparing it with the conventional vertical transwell plate. Therefore, a 200 μg/mL BSA solution was added into the upper chamber (chamber A) of a transwell plate or to the chamber A of the horizontal coculture plate. The BSA concentration in both chambers was measured after 1, 6 and 24 h of incubation. The ratio of detected BSA concentration in chamber B to that of chamber A reflects the diffusion capacity of both co-culture systems (supplemental Figure S5).

### 2.3. Flow Cytometric Analyses of M1/M2 Macrophages

After 5 days of JPCs/OBJPCs + M1/M2 coculture, macrophages were detached from the bottom of the horizontal plate by using TrypLE-Express, and M1 or M2 macrophages cell surface markers were detected by flow cytometry. After centrifugation (1400 rpm, 5 min) and removal of supernatants, cell pellets were resuspended in 10% Gamunex (human immune globulin solution, Talecris Biotherapeutics, Germany) and placed on ice for 15 min. Then, cells were incubated with specific antibodies (Biolegend, San Diego, CA, USA, Table 2) for 30 min in the dark. Subsequently, cells were washed twice with FACs buffer (PBS containing 0.1% BSA and 0.1% sodium azide) and measured by the Guava EasyCyte 6HT-2L flow cytometer (Merck Millipore, Germany). FlowJo software (Tree Star, Ashland, OR, USA) was used for data evaluation.

### 2.4. RNA Isolation and Quantitative Gene Expression Analyses in M1/M2 Macrophages

After 5 days of JPCs/OBJPCs + M1/M2 coculture, the total RNA from macrophages was isolated using the NucleoSpin RNA kit (Macherey-Nagel, Hoerd, France) as recommended by the manufacturer. RNA concentration and purity were photometrically measured and quantified by the NanoDrop One device (Thermo Fisher Scientific, Waltham, MA, USA). A total of 100 ng of RNA was used for cDNA synthesis according to the instructions of the SuperScript VILO Kit (Invitrogen, Thermo Fisher, Darmstadt, Germany). The real-time LightCycler System (Roche Diagnostics, Mannheim, Germany) was used to quantify mRNA expression levels. For the PCR reactions, DEPC-treated water, DNA Master SYBR Green I kit (Roche, Mannheim, Germany), and commercial primer kits from Search LC (Heidelberg, Germany) were used for 40 amplification cycles of the target DNA (CD163, CD209, TNF-α, CCL5, IL-6, IL-10 and CXCR4). The target gene transcript levels were normalized to those of the housekeeping gene GAPDH (Search LC, Heidelberg, Germany). For data evaluation and presentation, the gene ratio of the M1/M2 monoculture group was set to 1 (control), and x-fold induction indices relative to this control were calculated.

### 2.5. Analyses of Cytokine and Chemokine Release Using Proteome Profiler Arrays

In order to measure cytokines or chemokines secretion in the supernatants from M1/M2 macrophages after 5 days of coculture, human cytokine proteome profiler array kits (R&D Systems, Minneapolis, MN, USA) were used. Briefly, the proteome profiler membranes were blocked with array buffer for 1 h at RT and then incubated with sample supernatants and antibody mixtures overnight at 4 °C. After washing three times, the membranes were incubated with diluted streptavidin–HRP at RT for 30 min. After three washing steps again, the membranes were incubated with 1 mL of the chemiluminescent reagent mixture and exposed to the radiographic films for 10 min. After scanning of the developed x-ray films, data analysis of positive signals was quantified using the Image J software.

### 2.6. Immunofluorescence Staining 

After 5 days of coculture, M1 and M2 macrophages cultured in the horizontal plates were fixed with fixation buffer/paraformaldehyde (Biolegend, San Diego, CA, USA) for 30 min and incubated with 10% goat serum (Abcam, Cambridge, UK) for 1 h in order to block non-specific protein–protein binding. Then, M1 macrophages were incubated with the rabbit monoclonal anti-CD80 antibody (1:500 dilution)/mouse monoclonal anti-CD68 (1:100 dilution) antibody and M2 macrophages were treated with rabbit monoclonal anti-CD209 (1:100 dilution)/mouse monoclonal anti-CD68 (1:100 dilution) (Abcam, Cambridge, UK) antibodies overnight at 4 °C (antibodies were diluted in PBS containing 1% BSA and 0.1% Tween 20). Then, cells were incubated with secondary goat anti-mouse IgG Alexa Fluor^®^ 555 antibodies (1:500 dilution) and goat anti-rabbit IgG Alexa Fluor^®^ 488 antibodies (1:500 dilution) (Abcam, Cambridge, UK) for 1 h. Nuclei were counterstained with Hoechst 33342 (1 µg/mL, Promocell, Heidelberg, Germany) for 5 min. After rinsing in PBS, macrophages were visualized by an Observer Z1 fluorescence microscope (Zeiss, Oberkochen, Germany). Image J software was used to quantify the number of cell nuclei (Hoechst, blue fluorescence) and the number of CD80^+^/CD209^+^ cells (green fluorescence).

### 2.7. Macrophages DNA Replication Evaluation

To quantify the DNA synthesis of macrophages in the coculture system, the Click-iT™ EdU Alexa Fluor 488 flow cytometry assay kit (Thermo Fisher Scientific, Waltham, MA, USA) was used according to the manufacturer’s instructions. Therefore, 5 μM 5-ethynyl-2’-deoxyuridine (EdU, a thymidine analog) was added to the M1 or M2 macrophages chamber of the coculture plates on the second day of coculture. After 24 h of incubation, cells were analyzed by flow cytometry. The percentage of EdU-positive macrophages (click labeled with Alexa Fluor 488) was analyzed.

### 2.8. Cell Tracking

In order to observe intercellular communication from osteogenically induced JPCs to macrophages in our horizontal coculture system, we used nontransferable fluorescent dyes (CellTracker^TM^, Thermo Fisher Scientific, Waltham, MA, USA). Before the start of the coculture on day 7 (as outlined in Figure 1A), OBJPCs were labeled with red CMRA (20 µM) and M0 macrophages were labeled with green CMFDA (20 µM). After 30 min of incubation, the CellTracker solution was removed, and the CMRA-labeled OBJPCs and CMFDA-labeled M0 macrophages were cocultured under M1/M2 induction conditions for five days. Fluorescence images of macrophages were taken on day 12 and nuclei were stained with Hoechst 33342 (1 µg/mL, PromoCell, Heidelberg, Germany) for 5 min. After washing with PBS, microscopic pictures were taken.

### 2.9. Statistical Analysis

The data analysis for the performed measurements of three independent experiments was expressed as means ± standard error of means (mean ± SEM). A one-way analysis of variance (ANOVA) followed by Tukey’s multiple comparisons tests was used. All statistical analyses were carried out and visualized by using GraphPad Prism software (La Jolla, CA, USA). A value of *p* < 0.05 was considered as statistically significant.

## 3. Results

### 3.1. Phenotypic Changes of THP-1-Derived M1/M2 Macrophages Cocultured with JPCs/OBJPCs

In order to study the effects of JPCs/OBJPCs on THP-1-derived M1/M2 macrophages (M1/M2) polarization, the cell surface markers expression of M1 or M2 macrophages cocultured with or without JPCs/OBJPCs were measured by flow cytometry after 5 days of coculture. 

**Figure 2:**
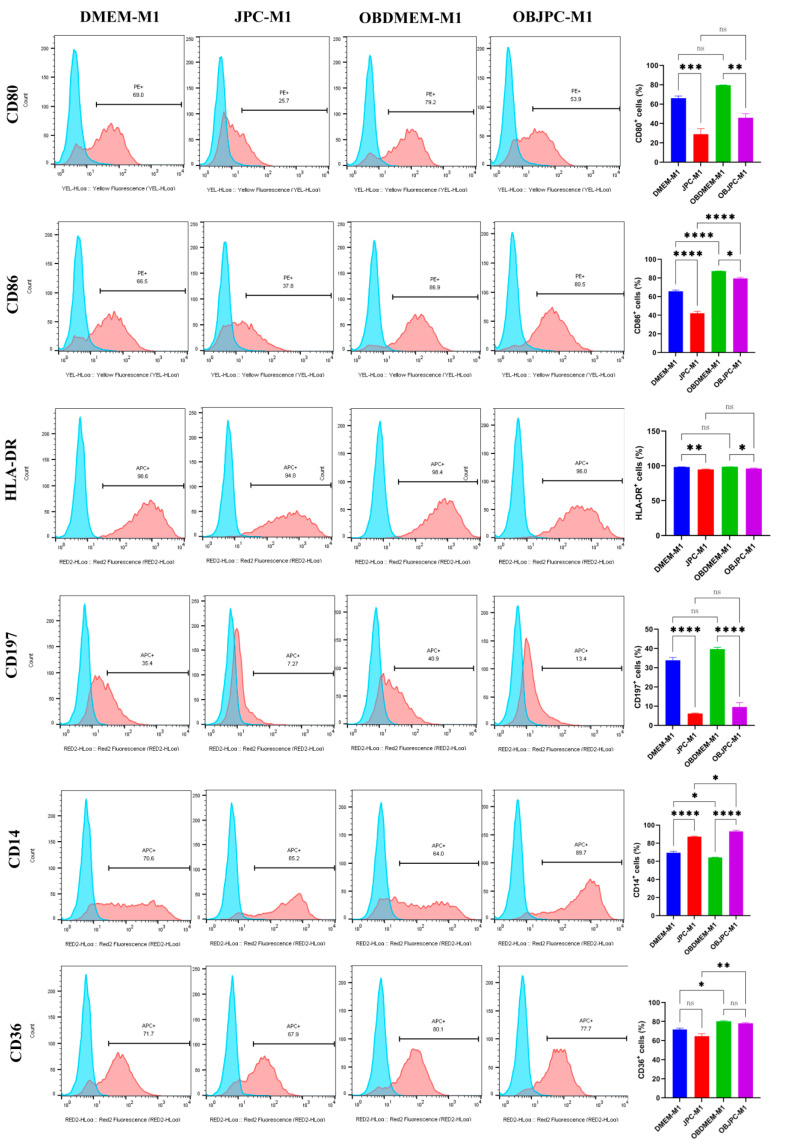
Representative single parameter histograms (blue peak: isotype control; red peak: targeted marker) and quantitative analysis of surface markers expression on M1 macrophages in the coculture system as detected by flow cytometry. Cell surface markers expression of CD80, CD86, HLA-DR, CD197, CD14 and CD36 in M1 macrophages cocultured with JPCs/OBJPCs under untreated (JPC-M1) and osteogenic (OBJPC-M1) conditions. Control groups were M1 macrophages cocultured with medium only (DMEM-M1 and OBDMEM-M1). Means ± SEM were calculated and compared using one-way ANOVA (n = 3, * *p <* 0.05, ** *p <* 0.01, *** *p <* 0.001, **** *p <* 0.0001). n.s. refers to non-significant.

The expression of surface markers on M1 macrophages are displayed in Figure 2. In the coculture groups (JPCs-M1 and OBJPCs-M1), CD80, CD86, HLA-DR and CD197 positive cells were significantly decreased compared to the monoculture control groups (DMEM-M1 or OBDMEM-M1). The differences in significance for CD80, CD86 and HLA-DR expression under osteogenic conditions were lower compared to those under untreated JPC conditions. In contrast, percentages of CD14 positive cells were significantly increased in the JPCs-M1 or OBJPCs-M1 groups compared to the DMEM-M1 or OBDMEM-M1 control groups. The detailed data are shown in Figure 2 and listed in Table 3.

**Figure 3:**
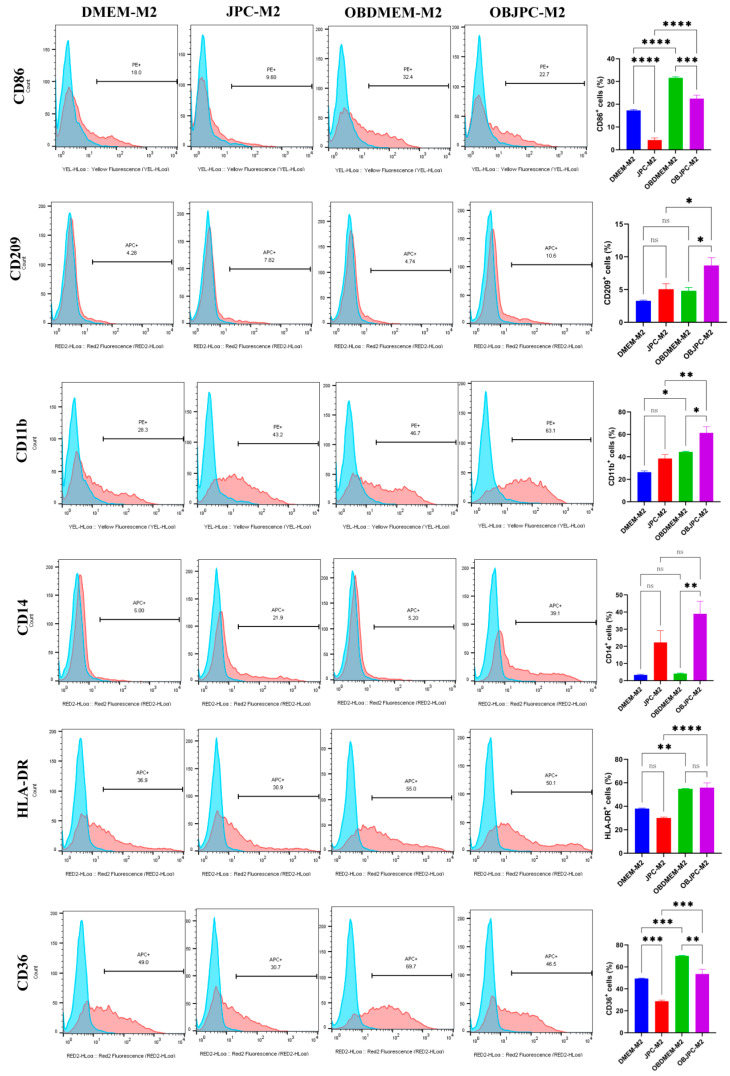
Representative single parameter histograms (blue peak: isotype control; red peak: targeted marker) and quantitative analysis of surface markers expression on M2 macrophages in the coculture system as detected by flow cytometry. Cell surface markers expression of CD86, CD209, CD11b, CD14, HLA-DR and CD36 in M2 macrophages cocultured with JPCs under untreated (JPC-M2) and osteogenic (OBJPC-M2) conditions. Control groups were M2 macrophages cocultured with medium only (DMEM-M2 and OBDMEM-M2). Means ± SEM were calculated and compared using one-way ANOVA (n = 3, * *p <* 0.05, ** *p <* 0.01, *** *p <* 0.001, **** *p <* 0.0001). n.s. refers to non-significant.

For flow cytometric analysis of cell surface markers expression on M2 macrophages (Figure 3), we found that percentages of CD209, CD11b and CD14 positive cells were significantly increased in the OBJPCs-M2 group compared to those detected in the OBDMEM-M2 control group. However, compared to the DMEM-M2 control group, the above markers just showed an increasing tendency in the JPC-M2 coculture group, but no significant difference was calculated. Furthermore, percentages of CD86 positive cells were significantly decreased in the JPC-M2 or OBJPC-M2 coculture group compared to those of the DMEM-M2 or OBDMEM-M2 control groups. The detailed data are shown in Table 4.

### 3.2. Gene Expression Analyses in M1/M2 Macrophages Co-Cultured with JPCs/OBJPCs

**Figure 4:**
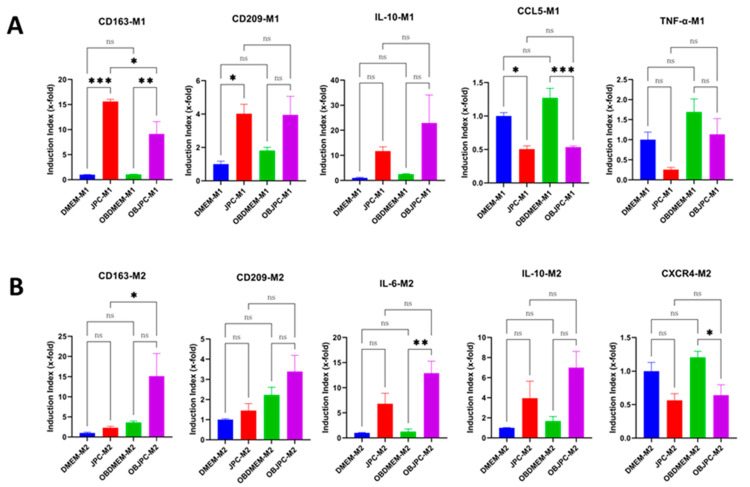
Quantitative gene expression of M1/M2 macrophages cocultured with untreated or osteogenically induced JPCs. (**A**): Gene expressions of CD163, CD209, IL-10, CCL5, and TNF-α in M1 macrophages cultivated in the DMEM-M1, JPC-M1, OBDMEM-M1 and OBJPC-M1 groups (n = 3, * *p <* 0.05, ** *p <* 0.01, *** *p <* 0.001). (**B**): Gene expressions of CD163, CD209, IL-6, IL-10 and CXCR4 in M2 macrophages cultivated in the DMEM-M2, JPC-M2, OBDMEM-M2 and OBJPC-M2 groups. Means ± SEM were calculated and compared using one-way ANOVA (n = 3, * *p <* 0.05, ** *p <* 0.01, *** *p <* 0.001). n.s. refers to non-significant.

In order to further evaluate the effects of untreated and osteogenically induced JPCs on macrophages polarization, the gene expression of CD163, CD209, TNF-α, CCL5, IL-6, IL-10 and CXCR4 in cocultured M1/M2 macrophages was measured by quantitative PCR. Results showed that CD163 and CD209 gene expression of the JPC-M1 group were significantly upregulated compared with that in the DMEM-M1 control group (CD163: DMEM-M1 1.000 ± 0.03116 versus JPC-M1 15.62 ± 0.42, *p <* 0.001; CD209: DMEM-M1 1.00 ± 0.18 versus JPC-M1 4.02 ± 0.57, *p <* 0.05). Similarly, CD163 gene expression in the OBJPC-M1 group was shown to be significantly upregulated compared to that in the OBDMEM-M1 group (CD163: OBDMEM-M1 1.03 ± 0.042 versus OBJPC-M1 9.12 ± 2.48, *p <* 0.01). In contrast, CCL5 gene expression in the JPC-M1 or OBJPC-M1 group was significantly downregulated compared with that in the DMEM-M1 or OBDMEM-M1 control group (CCL5: DMEM-M1 1.00 ± 0.05 versus JPC-M1 0.50 ± 0.05, *p <* 0.01; CCL5: OBDMEM-M1 1.27 ± 0.14 versus OBJPC-M1 0.53 ± 0.02, *p <* 0.001). Furthermore, compared to the monoculture control groups DMEM/OBDMEM-M1, TNF-α tended to be downregulated and IL-10 gene expression levels were shown to be upregulated in the JPCs/OBJPCs-M1 groups, respectively (Figure 4A).

Compared to the DMEM/OBDMEM-M2 monoculture control groups, the coculture groups JPCs/OBJPCs-M2 revealed an upregulated tendency for the CD163 or CD209 gene expression. IL-6 gene expression levels in the OBJPC-M2 coculture group were significantly upregulated compared to those detected in the OBMEM-M2 monoculture group (IL-6: OBDMEM-M2 1.22 ± 0.56 versus OBJPC-M2 12.93 ± 2.34, *p* < 0.01). Concerning the IL-10 gene expression, we detected a tendency for mRNA levels to be upregulated in the OBJPC-M2 coculture group compared to those from the OBDMEM-M2 monoculture group.

### 3.3. Cytokine and Chemokine Release Detection in Supernatants from M1/M2 Macrophages Cocultured with JPCs/OBJPCs by Proteome Profiler Arrays

**Figure 5:**
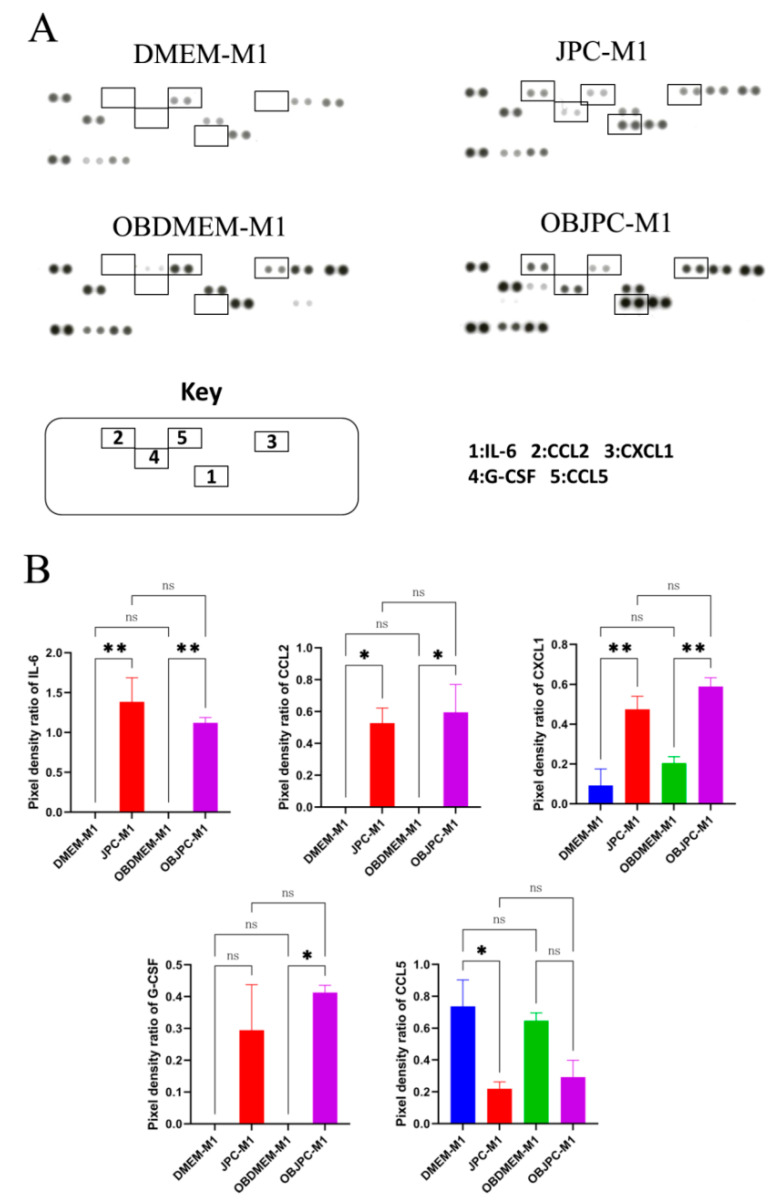
Protein expression analysis in supernatants of M1 macrophages cocultured with JPCs/OBJPCs as detected by proteome arrays and as quantified by Image J software. (**A**): Representative dot blots of different intensities detected after membrane incubation with supernatants from M1 macrophages (the rectangle-marked spots show significant differences between groups). (**B**): Quantification of pixel intensities for IL-6, CCL2, CXCL1, G-CSF and CCL5 protein expression. Means ± SEM were calculated and compared using one-way ANOVA (n = 3, * *p <* 0.05, ** *p <* 0.01). n.s. refers to non-significant.

**Figure 6:**
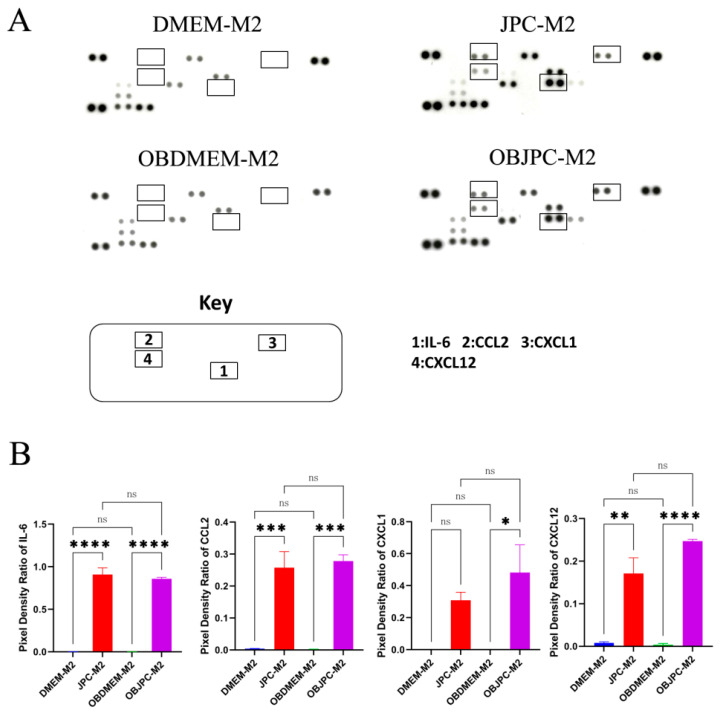
Protein expression analysis in supernatants of M2 macrophages cocultured with JPCs/OBJPCs as detected by proteome arrays and as quantified by Image J software. (**A**): Representative dot blots of different intensities detected after membrane incubation with supernatants from M2 macrophages (the rectangle-marked spots show significant differences between groups). (**B**): Quantification of pixel intensities for IL-6, CCL2, CXCL1 and CXCL12 protein expression. Means ± SEM were calculated and compared using one-way ANOVA (n = 3, * *p <* 0.05, ** *p <* 0.01, *** *p <* 0.001, **** *p <* 0.0001). n.s. refers to non-significant.

After coculturing JPCs/OBJPCs and M1/M2 macrophages for 5 days, the chemokine and cytokine productions in supernatants from M1/M2 macrophages were analyzed by proteome profiler arrays (Figure 5 and Figure 6). Representative parts of used membranes showing specific dot blot intensities after incubation with supernatants from M1 macrophages are shown in Figure 5A. Quantitative analyses of pixel densities revealed that protein levels of IL-6, CCL2 (MCP-1), CXCL1 (GRO-α), G-CSF and CCL5 (RANTES) changed considerably between monoculture and coculture groups. Compared to monoculture groups (DMEM-M1 or OBDMEM-M1), levels of IL-6, CCL2, CXCL1 and G-CSF were significantly upregulated in JPC-M1 and OBJPC-M1 groups. The detailed data are shown in Table 5.

Representative parts of array membranes showing the dot blots which represent cytokines/chemokines expression in supernatants of cocultured M2 macrophages are shown in Figure 6A. Quantitative analyses showed significantly increased levels of IL-6, CCL2 and CXCL12 protein expression in the coculture JPC-M2 group compared to the DMEM-M2 monoculture group. Similarly, compared to the OBDMEM-M2 monoculture control group, IL-6, CCL2, CXCL1 and CXCL12 levels were shown to be upregulated in the OBJPC-M2 coculture group. The detailed data are shown in Table 6.

### 3.4. Immunofluorescent Staining of M1/M2 Macrophages Cocultured with JPCs/OBJPCs

**Figure 7:**
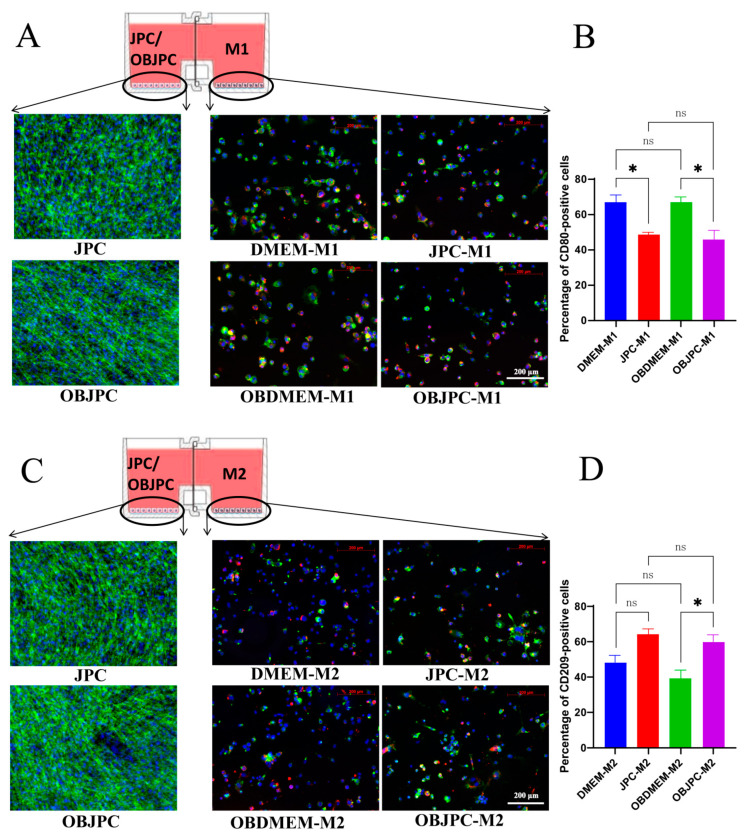
Immunofluorescence staining for JPCs/OBJPCs and M1/M2 macrophages. (**A**): Phalloidin PromoFluor-488 cytoplasm staining of JPCs/OBJPCs and CD68 Alexa Fluor 555 (red)/CD80 Alexa Fluor 488 (green) immunofluorescent staining for M1 macrophages; scale bar: 200 µm. (**B**): Semiquantitative analyses of the percentages of CD80-positive cells (n = 3 images per group, * *p <* 0.05). (**C**): Phalloidin PromoFluor-488 cytoplasm staining for JPCs/OBJPCs and CD68 Alexa Fluor 555 (red)/CD209 Alexa Fluor 488 (green) immunofluorescent staining for M2 macrophages; scale bar: 200 µm. (**D**): Analysis of the percentage of CD209-positive cells (n = 3 images per group, * *p <* 0.05). Blue cell nuclei were labeled with Hoechst 33342. n.s. refers to non-significant.

The immunofluorescent detection of CD68, CD80 expression for M1 cells or CD209 expression for M2 cells was used in order to confirm macrophages polarization. Additionally, cytoplasm staining using phalloidin Alexa Fluor 488 (green) was performed in order to visualize JPCs/OBJPCs co-cultivated with M1/M2 macrophages in the parallel compartment (Figure 7). Nuclei were counterstained with Hoechst 33342 (blue). The evaluation of immunofluorescent staining showed CD68 expression (red) in both M1 and M2 macrophages. Cytoplasm and nuclei staining of JPCs and OBJPCs showed very dense cell layers after co-culturing with M1/M2 macrophages for 5 days covering the bottom of the horizontal coculture plates.

For the detection of M1 macrophages after co-culturing with JPCs/OBJPCs, CD80 as a M1-specific cell surface marker was used. CD80 seemed to be expressed in both mono and cocultured M1 macrophages (green staining, Figure 7A). Quantitative analysis of the results showed that 48.42% of M1 macrophages cocultured with JPCs and 45.75% of M1 macrophages cocultured with OBJPCs expressed CD80. Compared to monocultured M1 cells of the DMEM-M1 group, 18.31% lower M1 cell numbers were CD80-positive in the JPC-M1 group. Similarly, 20.83% lower M1 cell numbers were CD80-positive in the OBJPC-M1 group compared to monocultured M1 cells in the OBDMEM-M1 group (Figure 7B).

After co-culturing M2 macrophages with JPCs/OBJPCs, the detection of the M2-specific surface marker CD209 was performed (green staining, Figure 7C). CD209 was found to be expressed in both mono- and cocultured M2 macrophages. Semiquantitative Image J analysis results showed that 63.91% of M2 macrophages cocultured with JPCs and 59.69% of M2 macrophages cocultured with OBJPCs expressed CD209 on their surface. Therefore, CD209-positive cell numbers were shown to be 16% higher in the JPC-M2 coculture than in the monocultured DMEM-M2 cell group. Additionally, 20.69% higher numbers of CD209-positive M2 macrophages were detected in the OBJPC-M2 coculture group compared to M2 numbers from the OBDMEM-M2 monoculture group (Figure 7D).

### 3.5. Proliferation Analysis of M1/M2 Macrophages Growing in Mono or Cocultures by Quantification of DNA Synthesis

**Figure 8:**
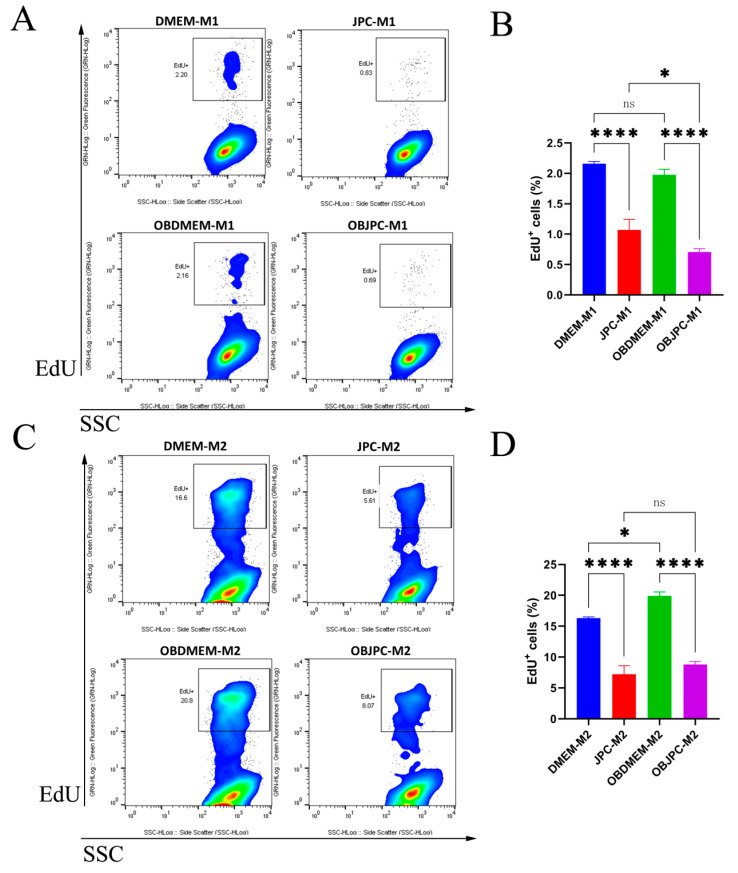
Flow cytometric measurements of DNA synthesis (EdU^+^ cells) in mono and cocultured M1/M2 macrophages. (**A**): Representative flow cytometric measurements of EdU^+^ M1 macrophages. (**B**): Quantification of EdU^+^ M1 macrophages in monocultures (DMEM-M1, OBDMEM-M1) and cocultures (JPC-M1, OBJPC-M1). (**C**): Representative flow cytometric measurements of EdU^+^ M2 macrophages. (**D**): Quantification of EdU^+^ M2 macrophages in monocultures (DMEM-M2, OBDMEM-M2) and cocultures (JPC-M2, OBJPC-M2). For all analyzed groups, 3 independent experiments were performed. Means ± SEM were calculated and compared using one-way ANOVA (n = 3, * *p <* 0.05, **** *p <* 0.0001). n.s. refers to non-significant.

On the third day of coculture, DNA synthesis of macrophages growing within the horizontal coculture system was determined by the quantification of EdU^+^ cells (Figure 8). In M1 cocultures, the percentages of EdU^+^ cells in the JPC- and OBJPC-M1 coculture groups were significantly reduced compared to those obtained in the monoculture groups DMEM- and OBDMEM-M1 (DMEM-M1 2.16 ± 0.04 versus JPC-M1 1.07 ± 0.18, *p <* 0.0001; OBDMEM-M1 2.00 ± 0.09 versus OBJPC-M1 0.71 ± 0.06, *p <* 0.0001, Figure 8A,B). In cocultures, the percentages of EdU^+^ M2 cells in the JPC- or OBJPC-M2 groups were significantly lower compared to those from the DMEM- or OBDMEM-M2 monoculture groups (DMEM-M2 16.30 ± 0.21 versus JPC-M2 7.24 ± 1.37, *p <* 0.0001; OBDMEM-M2 19.90 ± 0.64 versus OBJPC-M2 8.83 ± 0.45, *p* < 0.0001, Figure 8C,D).

### 3.6. Detection of the Endocytic Activity of M1/M2 Macrophages Cocultured with JPCs/OBJPCs

**Figure 9:**
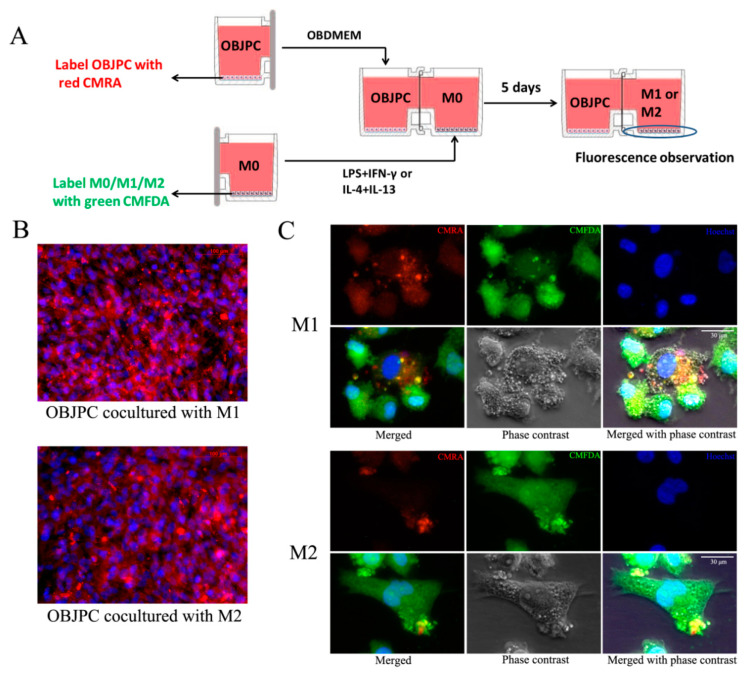
Detection of particles internalization in M1/M2 macrophages cocultured with OBJPCs within the horizontal coculture system by using the cell tracking method. (**A**): Schematic diagram of CMRA labeling of OBJPCs and CMFDA labeling of M0/M1/M2 macrophages. (**B**): Images of CMRA labeled OBJPCs after 5 days coculture with M1/M2 macrophages; scale bar: 100 µm. (**C**): Representative microscopic pictures of CMFDA labeled M1 and M2 macrophages (green) containing some CMRA-labeled particles from cocultured OBJPCs (red); scale bar: 30 µm. Blue cell nuclei were labeled with Hoechst 33342.

In order to evaluate the ability of particle internalization of M1/M2 macrophages cultured within the horizontal coculture system, we first labeled OBJPCs with the red CMRA fluorescence dye and M0/M1/M2 macrophages with the green CMFDA fluorescence dye (Figure 9A). After 5 days of coculture, we detected red-CMRA-labeled particles in the cytoplasm of M1 and M2 macrophages, but no green CMFDA in OBJPCs (Figure 9B). In Figure 9C, numerous red fluorescently labeled particles could be visualized in the cytoplasm of phagocytically active green-labeled M1-type macrophages. In contrast, a lower number of red-labeled particles were detected in the cytoplasm of green-labeled cocultured M2 macrophages (Figure 9C).

## 4. Discussion

In our previous study, we established protocols for the effective generation of phenotypically and morphologically different THP-1-derived M1 and M2 macrophages under hPL supplementation. Additionally, we examined the influence of JPCs on M1/M2 macrophage polarization when they were cocultured in direct contact and we showed evidence that JPCs inhibit M1 polarization [6]. In order to examine whether JPC effects on macrophages differentiation are based on soluble factors released by JPCs, an innovative horizontal coculture system was used in the present study. Compared with the traditional vertical transwell coculture system, the two cocultured cell types within the horizontal coculture system are cultivated under the same conditions: they are growing on the surface of the same material, the same volume of medium is added to both chambers and the semipermeable membrane between the chambers cannot be clogged by cells. These features can make sure an effective exchange of paracrine factors secreted by cocultured JPCs/OBJPCs and macrophages, as we could demonstrate by diffusion experiments using a defined BSA solution, as shown in the supplemental Figure S5. As an influential result of morphological changes, JPCs increased the elongation factor of cocultured M1 macrophages under osteogenic conditions (Figure S2).

Macrophages are professional antigen-presenting cells (APCs) able to activate T lymphocytes. Our experiments showed that almost all M1 macrophages expressed HLA-DR and less than 40% of generated M2 macrophages were HLA-DR-positive. This result is in line with findings from other studies [11]. Interestingly, osteogenic medium slightly enhanced HLA-DR expression. Additionally, we observed that JPCs/OBJPCs were able to effectively inhibit the expression of costimulatory molecules CD80 and CD86 on the surface of M1 macrophages. Another characteristic surface marker for M1 macrophages is CD197, also called C-C chemokine receptor type 7 (CCR7) [12]. CD197 binding to its ligands CCL19 and CCL21 can trigger migratory and inflammatory reactions [13,14]. CD197 expression on the surface of M1 macrophages was effectively suppressed by JPCs and OBJPCs. These results indicate that JPCs are able to reduce the ability of M1 macrophages to interact with or stimulate T cells under undifferentiated and osteogenic conditions.

Besides their antigen-presenting function, macrophages are able to ingest and process foreign materials, dead cells and debris, caused by microbial infection or tissue damage [15,16]. In this context, the expression of the hemoglobin–haptoglobin complex scavenger receptor (CD163) on the surface of macrophages is one of the major markers for the conversion of macrophages to the alternatively activated M2 phenotype and functions as an innate immune sensor for gram-positive and gram-negative bacteria [17,18,19]. Our data show that both JPCs and OBJPCs clearly increased gene expression of CD163 in cocultured M1 macrophages. CD163 activation can trigger anti-inflammatory IL-10 responses via the phosphatidylinositol-3 kinase-dependent Akt signaling [18]. The trend toward increasing IL-10 gene expression in the cocultured M1 and M2 macrophages groups supports the assumption of a M1 to M2 macrophages phenotype change under the secretory influence of JPCs.

CD36, a typical member of the scavenger receptor family, can form heteromultimeric signaling complexes with scavenger receptor cysteine family kinases and transmembrane proteins (such as toll-like receptor 2) [20,21,22]. CD36 expression on macrophages contributes to their characteristic phenotype by complexing with TLRs to potentiate inflammatory cytokine production [20]. In this study, JPCs were not able to decrease CD36 surface expression in M1 macrophages. Interestingly, both JPCs/OBJPCs significantly suppressed CD36 expression on M2 macrophages.

CD209 belongs to the C-type lectin receptor family and is expressed on the surface of macrophages as well as on dendritic cells. Increased expression of CD209 has been shown to be associated with the phenotype of M2 macrophages [23,24]. In our study, CD209 surface expression on M2 macrophages was relatively low. However, in the presence of OBJPCs, a clear upregulating tendency was observed in both M1 and M2 macrophages as shown by gene and protein expression analyses.

CCL5 (RANTES) is known as a chemoattractant for multiple immune cells, activating intracellular MAPK and NF-κB signaling pathways and promoting M1 macrophages polarization [25]. When M1 macrophages were cocultured with JPCs and OBJPCs, CCL5 gene and protein secretion levels were significantly decreased by JPCs, also reflecting an inhibitory effect of JPCs on M1 polarization.

**Figure 10:**
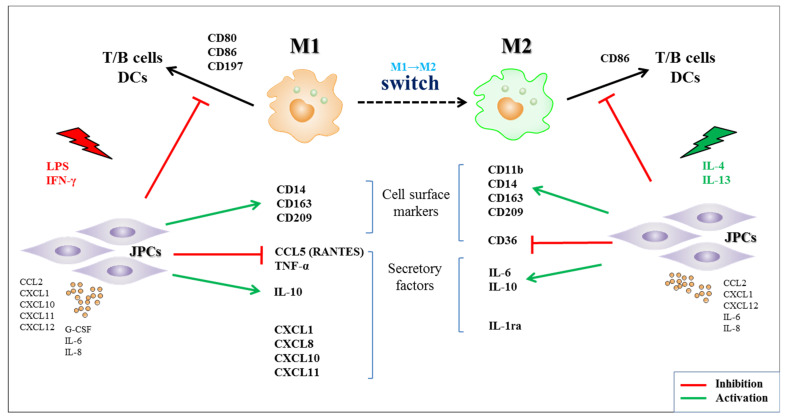
Summary of the interactions between JPCs and M1/M2 macrophages in the present study. We identified a variety of secretory factors which probably influence the JPC-induced interactions with macrophages and finally the M1→M2 macrophages switch (red interactions = inhibitory effects, green interactions = activating effects).

MSCs and inflammatory cells interact bidirectionally [26]. An acute inflammatory immune response is crucial at the onset of bone repair; the adaptive immune response comes into play during late bone remodeling. Inflammatory cells induce migration and differentiation of MSCs, initiating anabolic processes of bone regeneration. MSCs for their part can also regulate the secretion of proinflammatory cytokines. Specifically, under stress conditions, including microbial infection and sterile inflammation, MSCs can secrete chemokines (CXCL9, CXCL10, CXCL11) and cytokines (IL-6, IL-8, GM-CSF, G-CSF) to recruit neutrophils or lymphocytes, and enhance proinflammatory reactions [27,28,29]. Both, pro- and anti-inflammatory cytokines or cells, can also regulate MSCs properties. These effects were apparent in our OBJPC monoculture experiments as illustrated in the supplemental Figure S3 and Table S1. Activation with LPS/IFN-γ can stimulate secretion of the proinflammatory chemokines CXCL10 and CXCL11 by OBJPCs. In contrast, the IFN-γ inducible factors CXCL10 and CXCL11 were not released by JPCs under anti-inflammatory conditions. Proangiogenic IL-8 release by OBJPCs was also increased under proinflammatory conditions. It has been reported that amniotic MSCs contribute to wound healing by secreting IL-8 [30,31,32]. MSCs can release CCL2 (monocyte chemoattractant protein-1) to recruit monocytes/macrophages from the bone marrow and the splenic monocyte reservoir into inflamed tissues and promote wound healing [15,28,33,34,35]. Recent studies reported that CCL2 and CXCL12 (stromal cell-derived factor-1; SDF-1) chemokine secretion derived from MSCs cooperatively polarize IL-10-expressing tissue macrophages to mitigate gut injury [36]. In our study, OBJPCs constitutively produced CCL2 and CXCL12, and abundant CCL2 protein levels were detected in supernatants of M1/M2 macrophages in our cocultures. These results reveal a high chemotactic ability of OBJPCs on macrophages. Further, we demonstrated that JPCs/OBJPCs induced IL-10 gene expression in M1 and M2 macrophages, as shown in Figure 10.

CXCL1 (GRO-α), a chemokine known to be responsible for fine-tuning of neutrophil trafficking [37], was released by OBJPCs under both pro- and anti-inflammatory conditions. Both chemokines, CCL2 and CXCL1, can contribute to the repair of tissue injuries by attraction of macrophages and conversion of M1 to M2 phenotypes and further the controlled recruitment of neutrophils.

Furthermore, we hypothesize that high chemokine expression by JPCs results in attracting various immune cells to their proximity, where JPCs exert their immunosuppressive activities. When JPCs/OBJPCs were cocultured with M1/M2 macrophages, IL-6 levels were increased in supernatants from M1/M2 macrophages (Figure 5 and Figure 6). However, our experiments of OBJPCs monocultures showed evidence that the secreted IL-6 was constitutively produced by JPCs independent of exposure to pro- or anti-inflammatory cytokines (Figure S3). In our coculture experiments, we detected further increased gene expression levels of IL-10 in M1/M2 macrophages. These results suggest an activation of the alternative M2 phenotype through JPCs, confirming results from other studies reporting about monocyte (M0) polarization towards anti-inflammatory IL-10-producing M2 macrophages through IL-6-producing MSCs [28,38,39].

In addition, G-CSF, a classical regulator of neutrophil trafficking from bone marrow to blood [29,40], was abundantly produced by osteogenically induced JPCs under LPS/IFN-γ stimulation, and was also increased in M1 supernatants from coculture experiments. Interestingly, Wen and coauthors reported decreased M1/M2 ratios by G-CSF in both peripheral blood and bone marrow samples from healthy donors [41]. We assume that OBJPCs might secrete G-CSF under inflammatory stimulation in order to polarize M1 macrophages to the M2 phenotype in our coculture system.

For a better overview, we summarized the key interactions between JPCs and macrophages in this study and illustrated them in Figure 10. Under osteogenic conditions, JPCs stimulated by LPS + IFN-γ/IL-4 + IL-13 can secrete a variety of different cytokines/chemokines which may regulate macrophages polarization (Table S1). The expression of cell surface markers and the release of secretory factors (Figure S4) by macrophages were influenced by JPCs in the coculture and showed a M1 to M2 switch, which may further influence the T cells, B cells or DCs activation or maturation.

It has been stated that macrophages are mature differentiated cells that may possess self-renewal capacity similar to that of stem cells [42]. The anti-inflammatory cytokines IL-4 or IL-13 commonly used for M2 macrophages differentiation mediated proliferative activities of tissue-resident macrophages in several studies [43,44,45]. In our study, only around 2% of M1 macrophages were shown to be EdU^+^ but 17% of EdU^+^ M2 macrophages were detected. Flow cytometry analyses clearly revealed that both JPCs and OBJPCs suppressed the proliferative or self-renewal ability of M1 and M2 macrophages, thereby showing an immunosuppressive effect. The activation of toll-like receptors (TLRs) on macrophages by bacteria serves as a sensor for the internalization and phagosome maturation [46]. In our study, we found large numbers of particles dispersed in the cytoplasm, especially of M1 macrophages. Interestingly, detected “particles” looked like phagosomes and contained red fluorescence originating from red-labeled OBJPCs. We hypothesized that cocultured macrophages internalized substances from both chambers. However, whether this phagocytic phenomenon, and in particular the internalization of OBJPCs-derived substances, contributes to the regulation of macrophages polarization by JPCs requires further analysis.

## 5. Conclusions

By using the interactive horizontal coculture system, we systematically demonstrated that jaw periosteal cells secrete a plethora of factors promoting an effective conversion of macrophages from the classical M1 towards the alternative M2 phenotype. Additionally, untreated or osteogenically induced JPCs showed a clear potential to effectively inhibit self-renewal/proliferation activity of THP-1-derived M1 and M2 macrophages.

## Figures and Tables

**Table 1:** Used abbreviations for JPCs/OBJPCs coculture groups.

Abbreviation	Group	Cells	Medium	Reagents
DMEM	medium control	-	DMEM/F12/10% hPL	-
OBDMEM	osteogenic medium control	-	DMEM/F12/10% hPL	100 μM L-ascorbic acid + 10 mM β-glycerophosphate
JPC	cocultured untreated JPCs	JPCs	DMEM/F12/10% hPL	-
OBJPC	cocultured osteogenically induced JPCs	osteogenically induced JPCs	DMEM/F12/10% hPL	100 μM L-ascorbic acid + 10 mM β-glycerophosphate

**Table 2:** List of antibodies used for flow cytometry.

Human Antigen	Clone	Isotype Control	Conjugate
CD80	2D10	IgG1	PE
CD86	BU63	IgG1	PE
CD11b	ICRF44	IgG1	PE
HLA-DR	L243	IgG2a	APC
CD209	9E9A8	IgG2a	APC
CD197	G043H7	IgG2a	APC
CD36	5–271	IgG2a	APC
CD14	M5E2	IgG2a	APC

**Table 3:** Percentages of positive M1 macrophages for the listed CD markers cocultured with undifferentiated and osteogenically induced JPCs (JPCs/OBJPCs).

Markers	DMEM-M1	JPC-M1	OBDMEM-M1	OBJPC-M1
CD80	66.12 ± 2.24	28.87 ± 5.84 ^a^	79.46 ± 0.29	45.91 ± 4.18 ^b^
CD86	65.68 ± 1.40	42.05 ± 2.14 ^a^	87.26 ± 0.22	79.45 ± 1.22 ^b^
HLA-DR	98.33 ± 0.25	94.98 ± 0.48 ^a^	98.57 ± 0.08	96.18 ± 0.70 ^b^
CD197	33.87 ± 1.47	6.15 ± 0.31 ^a^	39.61 ± 0.97	9.56 ± 2.35 ^b^
CD14	69.45 ± 1.63	87.22 ± 0.54 ^a^	64.01 ± 0.48	93.20 ± 1.16 ^b^
CD36	71.53 ± 1.80	64.59 ± 2.86	80.42 ± 0.77	78.21 ± 0.84

^a^ Significant differences (*p <* 0.05) were detected between the DMEM-M1 monoculture control and the JPC-M1 coculture groups; ^b^ significant differences (*p <* 0.05) were detected between the OBDMEM-M1 monoculture control and the OBJPC-M1 coculture groups.

**Table 4:** Percentages of positive cells for the listed CD markers of M2 macrophages cocultured with JPCs/OBJPCs.

Markers	DMEM-M2	JPC-M2	OBDMEM-M2	OBJPC-M2
CD86	17.33 ± 0.43	4.29 ± 0.93 ^a^	31.54 ± 0.53	22.51 ± 1.50 ^b^
CD209	3.29 ± 0.10	5.06 ± 0.83	4.81 ± 0.51	8.68 ± 1.19 ^b^
CD11b	26.37 ± 1.15	38.39 ± 3.53	44.23 ± 0.73	61.39 ± 5.43 ^b^
CD14	3.37 ± 0.34	22.30 ± 6.88	4.16 ± 0.26	39.01 ± 7.20 ^b^
HLA-DR	37.95 ± 0.51	29.97 ± 0.79	54.74 ± 0.49	55.88 ± 4.02
CD36	49.51 ± 0.33	28.78 ± 0.97 ^a^	70.05 ± 0.43	53.63 ± 4.11 ^b^

^a^ Significant differences (*p <* 0.05) were detected between DMEM-M2 monoculture control and JPC-M2 coculture groups; ^b^ significant differences (*p <* 0.05) were detected between the OBDMEM-M2 monoculture control and the OBJPC-M2 coculture groups.

**Table 5:** The pixel density ratio of cytokines/chemokines in M1 macrophages supernatants in the coculture system.

Cytokine/Chemokine	DMEM-M1	JPC-M1	OBDMEM-M1	OBJPC-M1
IL-6	0.00 ± 0.00	1.39 ± 0.30 ^a^	0.00 ± 0.00	1.12 ± 0.07 ^b^
CCL2	0.00 ± 0.00	0.53 ± 0.10 ^a^	0.00 ± 0.00	0.60 ± 0.17 ^b^
CXCL1	0.09 ± 0.08	0.48 ± 0.06 ^a^	0.20 ± 0.03	0.59 ± 0.04 ^b^
G-CSF	0.00 ± 0.00	0.29 ± 0.14	0.00 ± 0.00	0.41 ± 0.02 ^b^
CCL5	0.74 ± 0.17	0.22 ± 0.04 ^a^	0.65 ± 0.05	0.30 ± 0.11

^a^ Significant differences (*p <* 0.05) were detected between DMEM-M1 monoculture control and JPC-M1 coculture groups; ^b^ significant differences (*p <* 0.05) were detected between OBDMEM-M1 monoculture control and OBJPC-M1 coculture groups.

**Table 6:** The pixel density ratio of proteins in M2 macrophages supernatants in the coculture system.

Cytokine/Chemokine	DMEM-M2	JPC-M2	OBDMEM-M2	OBJPC-M2
IL-6	0.00 ± 0.00	0.91 ± 0.08 ^a^	0.00 ± 0.00	0.86 ± 0.02 ^b^
CCL2	0.00 ± 0.00	0.26 ± 0.05 ^a^	0.00 ± 0.00	0.28 ± 0.02 ^b^
CXCL1	0.00 ± 0.00	0.31 ± 0.05	0.00 ± 0.00	0.48 ± 0.17 ^b^
CXCL12	0.01 ± 0.00	0.17 ± 0.04 ^a^	0.00 ± 0.00	0.25 ± 0.00 ^b^

^a^ Significant differences (*p <* 0.05) were detected between DMEM-M2 control and JPC-M2 coculture groups; ^b^ significant differences (*p <* 0.05) were detected between OBDMEM-M2 monoculture and OBJPC-M2 coculture groups.

## Data Availability

The data that support the findings of this study are available from the corresponding author upon reasonable request.

## Supplementary Materials

The following are available online at https://www.mdpi.com/article/10.3390/biomedicines9121753/s1. Figure S1. Analysis of the osteogenic differentiation capacity of used jaw periosteal cells derived from 3 donors, as detected by the Alizarin-staining of calcium phosphate precipitates. Figure S2. Cell morphology and quantification of the elongation factor of mono- and cocultured M1/M2 macrophages. Figure S3. Protein expression analyses in supernatants from monocultured osteogenically induced JPCs. Figure S4. Comparison of cytokines/chemokines secretion in the supernatants of monocultured M1 and M2 macrophages under normal or osteogenic conditions. Figure S5. Comparison of the BSA diffusion capacity within the conventional transwell and the horizontal coculture system after 1, 6 and 24 h of incubation. Table S1: Secretion of OBJPCs in monocultures.
