# Supplementary material for: Analysis of the Influence of Jaw Periosteal Cells on Macrophages Phenotype Using an Innovative Horizontal Coculture System
Source: Biomedicines. 2021 Nov 24;9(12):1753. doi: 10.3390/biomedicines9121753 (PMC8698728; doi:10.3390/biomedicines9121753)
Supplement: Supplementary file 1 [file biomedicines-09-01753-s001.zip › biomedicines-1426210-supplementary.pdf]

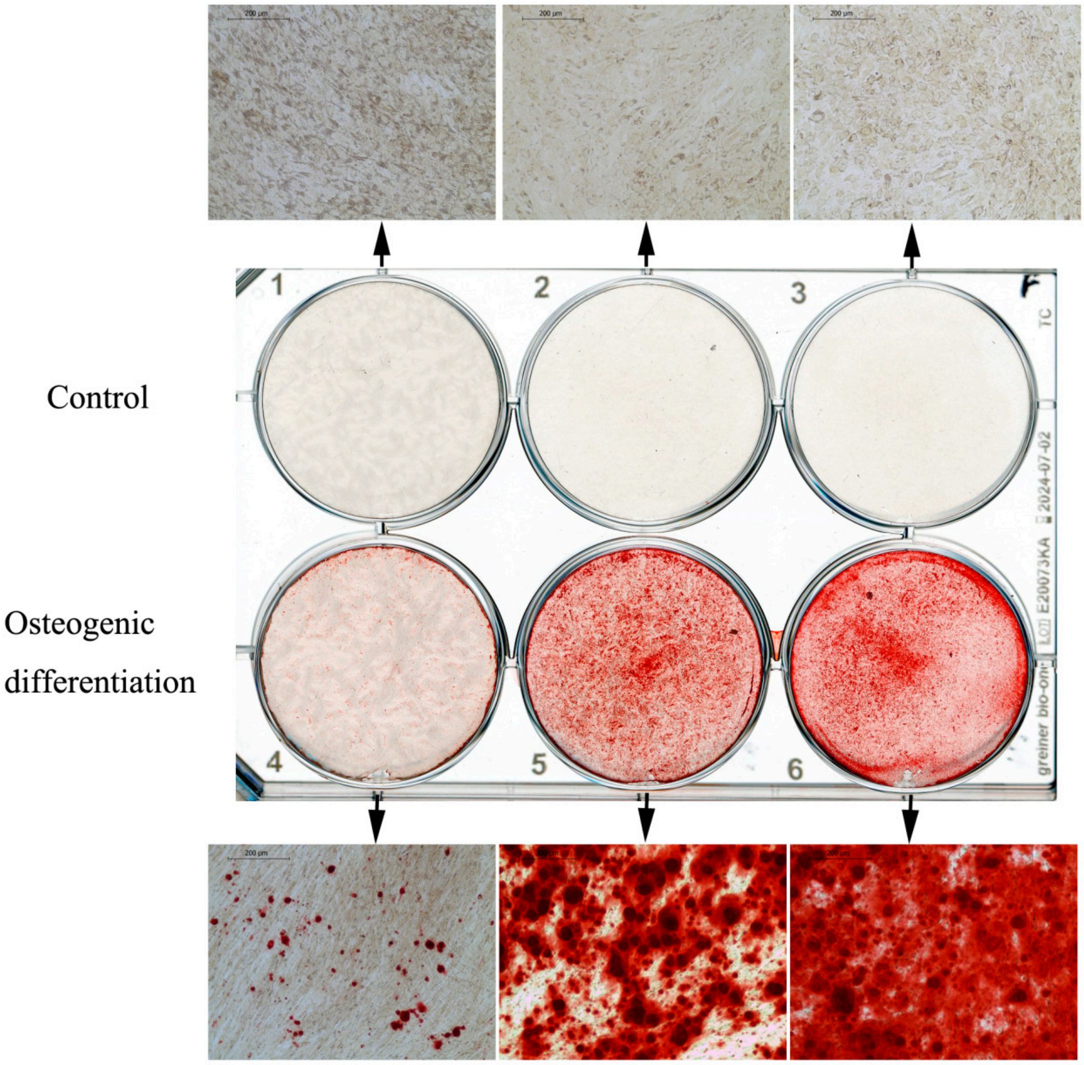

Figure S1. Analysis of the osteogenic differentiation capacity of used jaw periosteal cells derived from 3 donors, as detected by the Alizarin staining of calcium phosphate precipitates (red); scale bar: 200 µm.

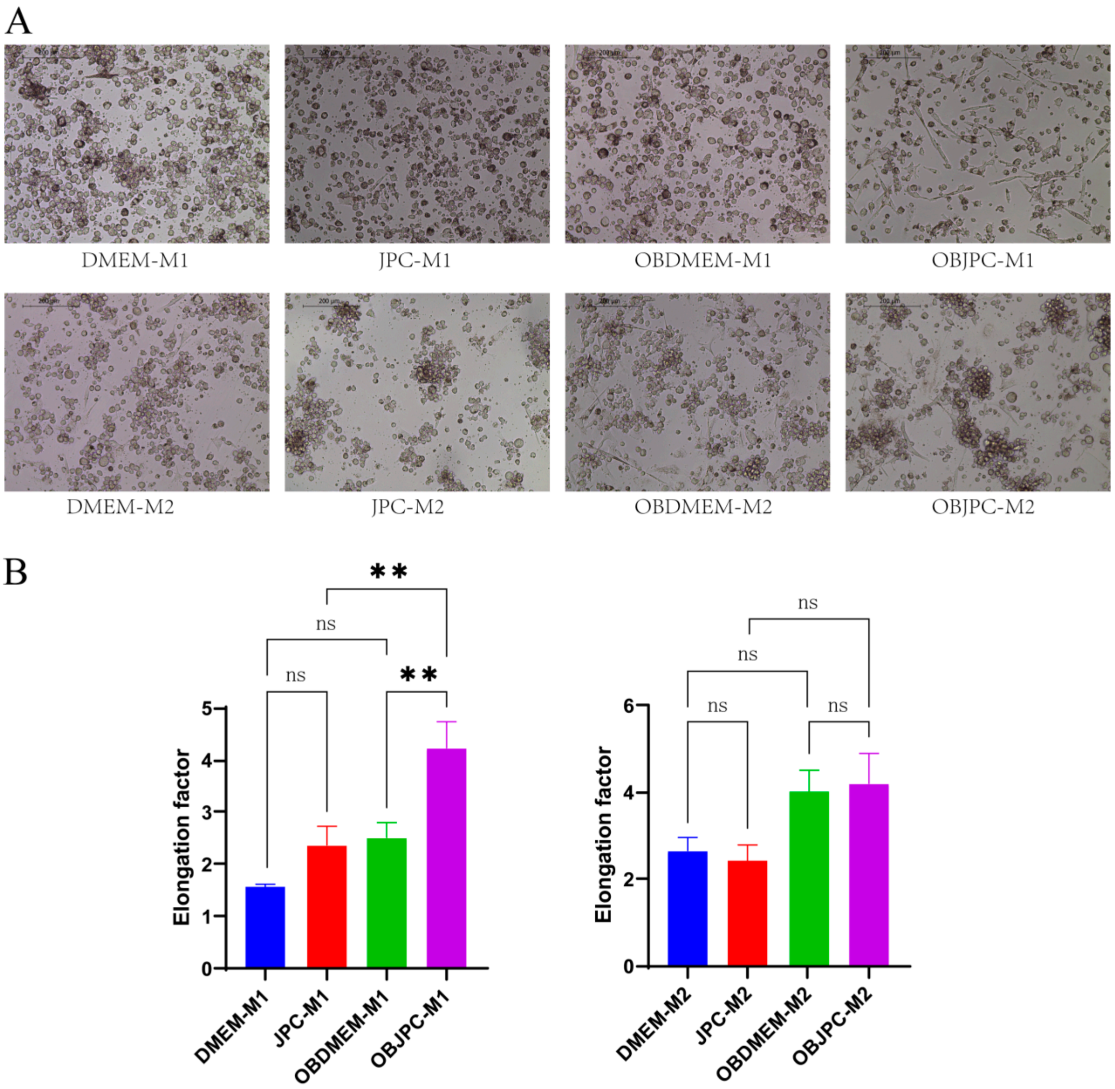

Figure S2. Cell morphology and quantification of the elongation factor of mono- and cocultured M1/M2 macrophages. A: Representative phase contrast microscopic pictures of mono- (DMEM, OBDMEM groups) and cocultured (JPC, OBJPC groups) M1 and M2 macrophages cultivated within the coculture system; scale bar: 200 µm. B: Analysis of the elongation factor of mono- and cocultured M1 and M2 macrophages (n=30 cells per group. ***$p<0.01$)

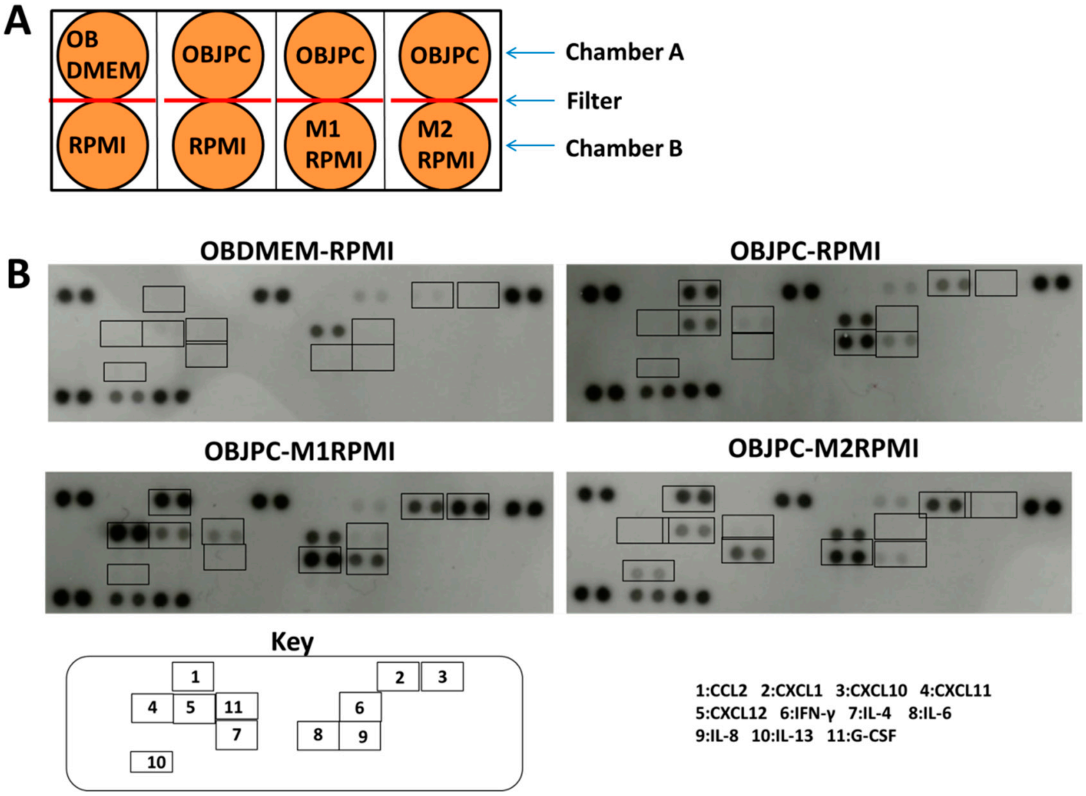

Figure S3. Protein expression analyses in supernatants from monocultured osteogenically induced JPCs (OBJPC). A: Layout of the OBJPC monoculture experiments (OBDMEM: cell-free OBDMEM medium; OBJPC: osteogenically induced JPCs; RPMI: cell-free RPMI medium; M1RPIMI: cell-free RPMI medium + LPS + IFN-γ; M2RPMI: cell-free RPMI medium + IL-4 + IL-13). B: Images of proteome membranes incubated with supernatants collected from chamber B (the rectangle marked the spots which were differentially expressed proteins between groups).

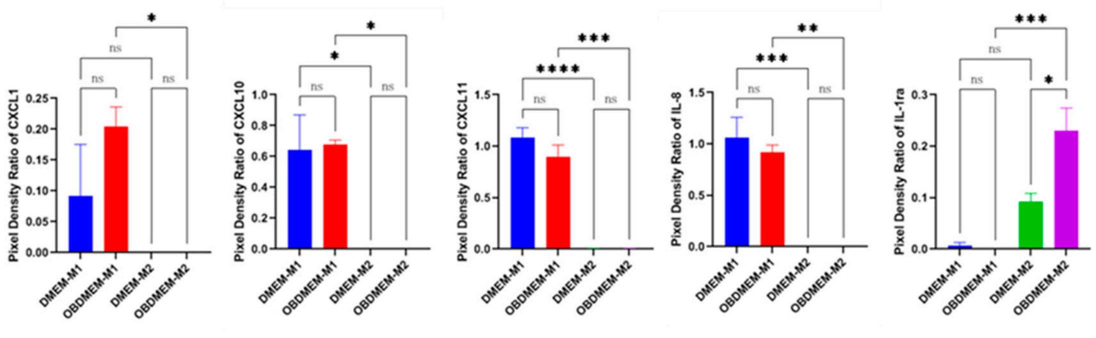

Figure S4. Comparison of cytokines/chemokines secretion in the supernatants of monocultured M1 and M2 macrophages under normal (DMEM) or osteogenic (OBDMEM) conditions (n=3, *p < 0.05, **p < 0.01, ***p < 0.001, ****p < 0.0001).

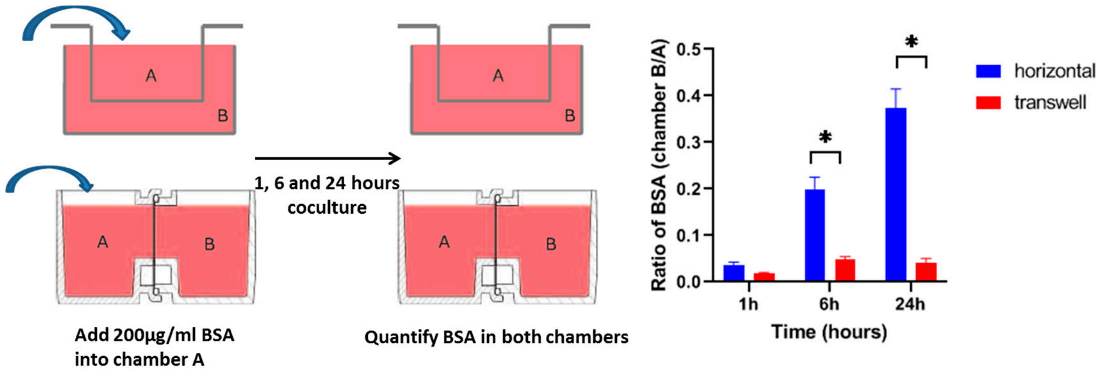

Figure S5. Comparison of the BSA diffusion capacity within the conventional transwell and the horizontal coculture system after 1, 6 and 24 hours of incubation. The ratio of quantified BSA (chamber B/chamber A) in the horizontal coculture plate was significantly higher than that detected in the transwell plate.

Table S1: Secretion of OBJPCs in mono-cultures. Secretion of OBJPCs cultivated as monocultures in the horizontal coculture system. OBJPCs were cocultured under M1 (LPS/IFN-γ) or M2 (IL-4/IL-13) conditions and cytokines/chemokines expression patterns were analyzed by human cytokine proteome arrays.

<table>
<tr><th>Groups</th><th>M1 culture conditions (LPS/IFN-γ)</th><th>M2 culture conditions (IL-4/IL-13)</th></tr>
<tr><td rowspan="8">Cytokines/ Chemokines</td><td colspan="2">CCL2/MCP-1</td></tr>
<tr><td colspan="2">CXCL1/GROα</td></tr>
<tr><td>CXCL10/IP-10</td><td></td></tr>
<tr><td>CXCL11/I-TAC</td><td></td></tr>
<tr><td colspan="2">CXCL12/SDF-1</td></tr>
<tr><td>G-CSF</td><td></td></tr>
<tr><td colspan="2">IL-6</td></tr>
<tr><td>IL-8 ^{high}</td><td>IL-8 ^{low}</td></tr>
</table>
